# Identification of radiomic biomarkers in a set of four skeletal muscle groups on Dixon MRI of the NAKO MR study

**DOI:** 10.1186/s12880-023-01056-9

**Published:** 2023-08-08

**Authors:** Marc Fischer, Thomas Küstner, Sofia Pappa, Thoralf Niendorf, Tobias Pischon, Thomas Kröncke, Stefanie Bette, Sara Schramm, Börge Schmidt, Johannes Haubold, Felix Nensa, Tobias Nonnenmacher, Viktoria Palm, Fabian Bamberg, Lena Kiefer, Fritz Schick, Bin Yang

**Affiliations:** 1 Institute of Signal Processing and System Theory, University of Stuttgart, Stuttgart, Germany; 2 Medical Image and Data Analysis (MIDAS.lab), University Hospital Tübingen, Tübingen, Germany; 3 Section on Experimental Radiology, University Hospital Tübingen, Tübingen, Germany; 4 Berlin Ultrahigh Field Facility (B.U.F.F.), Max-Delbrück-Center for Molecular Medicine, Berlin, Germany; 5 Department of Diagnostic and Interventional Radiology, University Hospital Augsburg, Augsburg, Germany; 6 Centre for Advanced Analytics and Predictive Sciences (CAAPS), University Augsburg, Augsburg, Germany; 7 Institute for Medical Informatics, Biometry and Epidemiology, Essen University Hospital, Essen, Germany; 8 Essen University Hospital, Essen, Germany; 9 University Hospital Heidelberg, Heidelberg, Germany; 10 University Medical Center Freiburg, Freiburg, Germany; 11 Department of Radiology, University Hospital Tübingen, Tübingen, Germany

**Keywords:** German national cohort, Radiomics, Texture feature analysis, Magnetic resonance imaging

## Abstract

In this work, we propose a processing pipeline for the extraction and identification of meaningful radiomics biomarkers in skeletal muscle tissue as displayed using Dixon-weighted MRI. Diverse and robust radiomics features can be identified that may be of aid in the accurate quantification e.g. varying degrees of sarcopenia in respective muscles of large cohorts. As such, the approach comprises the texture feature extraction from raw data based on well established approaches, such as a nnU-Net neural network and the Pyradiomics toolbox, a subsequent selection according to adequate conditions for the muscle tissue of the general population, and an importance-based ranking to further narrow the amount of meaningful features with respect to auxiliary targets. The performance was investigated with respect to the included auxiliary targets, namely age, body mass index (BMI), and fat fraction (FF). Four skeletal muscles with different fiber architecture were included: the mm. glutaei, m. psoas, as well as the extensors and adductors of the thigh. The selection allowed for a reduction from 1015 available texture features to 65 for age, 53 for BMI, and 36 for FF from the available fat/water contrast images considering all muscles jointly. Further, the dependence of the importance rankings calculated for the auxiliary targets on validation sets (in a cross-validation scheme) was investigated by boxplots. In addition, significant differences between subgroups of respective auxiliary targets as well as between both sexes were shown to be present within the ten lowest ranked features by means of Kruskal-Wallis H-tests and Mann-Whitney U-tests. The prediction performance for the selected features and the ranking scheme were verified on validation sets by a random forest based multi-class classification, with strong area under the curve (AUC) values of the receiver operator characteristic (ROC) of 73.03 ± 0.70 % and 73.63 ± 0.70 % for the water and fat images in age, 80.68 ± 0.30 % and 88.03 ± 0.89 % in BMI, as well as 98.36 ± 0.03 % and 98.52 ± 0.09 % in FF.

## Introduction

Radiomics has been an integral part in bringing omics to medical imaging data. Radiomics can be considered part of the holistic imiomics concept [1], which has the goal to leverage available imaging information alongside non-imaging data. By identification of extracted features from imaging data, quantifying phenotypic characteristics becomes possible, which allows to unveil correlations between raw data, relevant subgroups and their clinical outcome. These include the vast range of oncology applications [2], including chemotherapy response prediction [3], lung cancer screening [4], breast cancer differentiation [5] or osteoporosis [6]. The radiomics tools can also be used in conjunction with classical measures such as the fat infiltration [7] or muscle volumes [8].

Recently, sarcopenia was investigated [9–11] by aid of such radiomic features. Sarcopenia is a degenerative illness resulting in the loss of muscle mass (atrophy) and strength especially prevalent with increasing age or restricted mobility [12]. Modern imaging technologies allow for the visualization of respective (muscle) tissue and its degeneration in different ways [13, 14]. Thus, an accurate quantification based on radiomic (texture) features that gather information of the muscles, which may be difficult to identify from inspection of the gray values alone, alongside morphological (shape) characteristics becomes possible for specific muscles with recent (radiomics and segmentation) tools. These features may enable quantifying underlying deviations from the norm present within respective muscles. Subsequently, quantified deviations could be made usable as indicators in subsequent radiological analyses [14]. As such, varying degrees of severity of sarcopenia could be analyzed in large population-based cohorts as well as specific muscles of individual patients. Thus, analyzing the raw imaging data in an automated way becomes a mandatory and necessary step that allows for a statistical characterisation and gives way to personalized precision medicine.

Further, the robustness and reliability of such standardized texture features [15–19] were investigated and consensus proposals on their inclusion into clinical trials were developed [20]. Several MRI protocols have been investigated thoroughly, with respect to their nature of inhomogeneities, texture differences and relative signal intensities [21–26] and their prognostic value [27]. In this regard, there have also been efforts in providing intensity standardization for quantitative imaging [28].

Besides these applications and considerations, several methodical advancements regarding the selection process of important texture features have been evaluated in [29] for lung cancer tumors in CT images. Sugai et al. relied on the PyRadiomics toolbox [30] to calculate and extract standardized quantitative features based on imaging raw data. Subsequently features were selected via different selection methods (test-retest on a specific longitudinal dataset or correlation analysis). Remaining features where used in a lasso cox regression model in a five-fold cross-validation setting to obtain radiomic model estimators. Kim et al. [11] investigated the use of several different machine learning methods (suppport vector machines, random forests, extreme gradient boosting) to assess the usability of PyRadiomics texture features for the detection of sarcopenia in CT images. In addition, the combination with recent deep learning tools, such as GAN-based super-resolution [31] or entirely deep-learning-based radiomics pipelines [32] were explored.

In recent years, large population-based cohorts, such as the German National Cohort (GNC) [33] have been established with the goal to enable researchers to identify meaningful characteristics from non-invasive radiological imaging data. While most of the work considered under the umbrella of radiomics has been focused on different tumor types, we want to apply the same principles to MRI data of pre-dominantly healthy muscle tissue. This study is designed as a precursor for the identification of radiomic features with the long-term aim to identify a prognostic marker for the progression of sarcopenia. To this end, we

- propose a general processing pipeline to identify robust radiomics features for muscle tissue in large MRI cohorts comprised of 10672 eligible subjects,
- show that a diverse set of selected features can be ranked and thereby ordered based on their relative importance,
- analyze important features for sarcopenia by means of their statistical differences between subgroups based on available auxiliary information such as age and sex.

This pipeline is implemented and studied to serve as as a base for future investigation in the differentiability of texture features to detect varying degrees of sarcopenia. Thus, we want to identify diverse and robust features based on auxiliary information. The pipeline itself consists of two parts: a feature generation, and a feature selection. In the generation step, an automated muscle segmentation is followed by a feature extraction. The extraction incorporates the aforementioned established PyRadiomics toolbox [30]. In the feature selection part, several selection criteria as well as a suitable ranking scheme are integrated. Relevant processing steps from the literature are implemented into the pipeline to enable radiomic analyses.

## Methods

The proposed processing pipeline generates and selects radiomics features from MR imaging data. The used two-point Dixon MR data is described in Medical data section. The pipeline itself consists of two parts; the first part (Feature generation section) is comprised of an automated segmentation of muscular tissue groups and a subsequent extraction of textural and auxiliary information: age, body mass index (BMI), fat fraction (FF). In turn, the auxiliary information serves as surrogate targets in the second part (Feature selection section), in which a multi-step selection is performed to gain promising features. Thereby, we generate radiomics features of multiple muscle regions, allowing for further analyses of their characteristics and to identify potential biomarkers. In this work, we include four different muscle groups: the gluteus, the psoas as well as the extensors and the adductors of the thigh. We analyze these muscles by comparison across different subgroups, namely the subject’s age, their BMI (weight/height^2^), and the FF of each respective muscle (proportion of the acquired signal derived from Dixon water and fat images). In addition, statistical differences between those subgroups reflected in selected features were investigated. Here, we introduce a ranking scheme to identify the most important features by relying on multiple validation folds and targets. The proposed framework will be made publicly available: https://github.com/lab-midas/muscle_texture.

### Medical data

We focus our investigation on the whole-body T1-weighted dual echo gradient echo (GRE) MRI sequence (two-point Dixon) of the GNC (matrix size 240 x 320, resolution $1.2\textrm{mm}\times 1.2\textrm{mm}$, slice thickness 3mm, echo times 1.23/2.46ms, repetition time 4.36, flip angle $9^{\circ }$, band width 680Hz/pixel) acquired on 3T clinical MRI scanners (Magnetom Skyra, Siemens Healthineers, Erlangen, Germany) [34]. 11026 subjects were made available to us (baseline) with the total number of subjects expected to include 30000 imaging datasets [35]. The participants were between the age of 20 and 69 years and selected at random from the general German population. For the 11026 subjects, imaging data and anthropometric information (age, gender, weight, height) were available and were considered for the generation and selection of radiomics features. In the end, 10672 subjects, 5484 $(51.39\%)$ male and 5188 $(48.61\%)$ female, were suitable to be processed in the texture feature generation and selection. In Feature generation section further details on the auxiliary targets and the exclusion of ineligible subjects are provided. The water and fat images (contrasts) of the two-point Dixon scans were both considered for feature generation.

### Radiomics pipeline

**Fig. 1:**
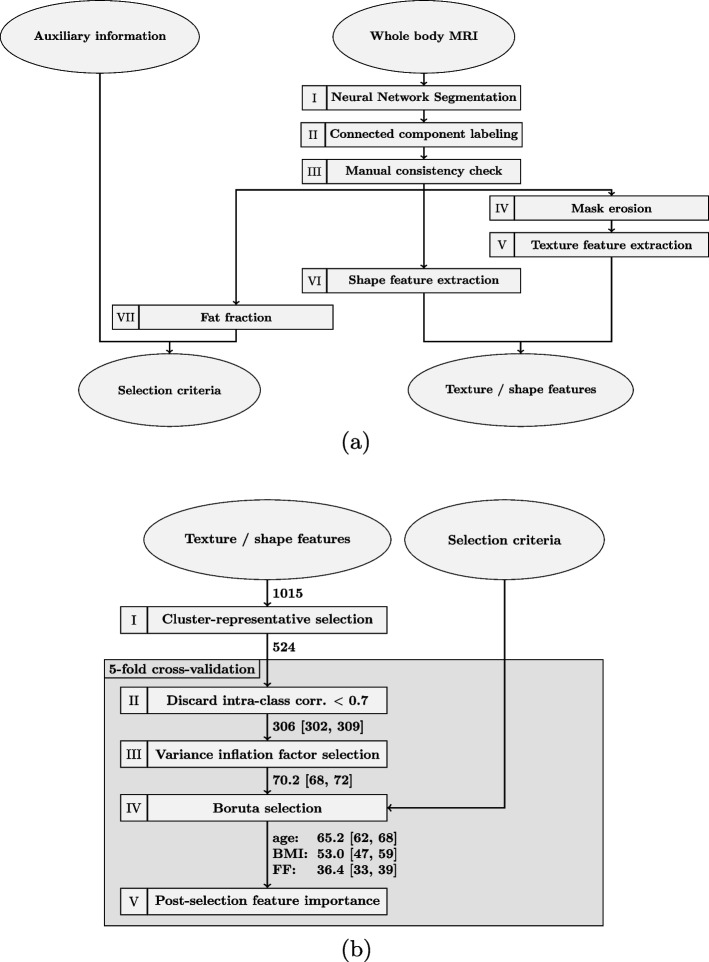
Proposed radiomics pipeline. **a** Radiomics feature generation pipeline. I: Deep learning based segmentation, II: Connected component labeling removing isolated mask elements, III: Manual consistency check of segmentation masks, IV: Mask erosion to remove boundary elements, V: Texture feature extraction of each muscle group, VI: Shape feature extraction of each muscle group, VII: fat fraction calculation based on water and fat contrast per muscle mask. **b** Radiomics feature selection pipeline with five-fold cross-valdiation for steps II-V. I: Cluster representative identification based on correlation values, II: Discarding features with low mean left and right muscle correlation values, III: Variance inflation factor calculation and iterative feature removal, IV: Heuristic Boruta selection to estimate feature importance with respect to surrogate targets body mass index (BMI), age and fat fraction (FF), V: Permutation importance calculation across folds for identification of ranking of selected features. Numbers on the arrows indicate the remaining amount of texture features after each selection step

The goal of the implemented radiomics pipeline is to provide a reproducible selection of robust radiomics features for the data. We achieve this by several generation and selection steps, before performing a final selection of a limited amount of features based on a proposed ranking scheme. The pipeline operates on the individual muscles and comprises an automatic segmentation, the feature extraction, several feature selections and the feature importance ranking. The ranking scheme can be applied to individual muscles or jointly to all available muscle groups together. The whole pipeline from generation over selection and importance calculation is introduced in the following sub-chapters and illustrated in Fig. 1.

#### Feature generation

##### Neural Network Segmentation

To extract relevant muscle groups, a deep learning network - the nnUnet [36] - was implemented as a robust method to handle different image sizes and provide sufficient automated segmentation performance. The process is described in more detail in [37]. For ease of application, two networks have been trained. One for the gluteus and psoas compartments, and one for the anterior (extensors) and posterior (adductors) muscle compartments. Each network predicts four categories due to a further separation of each muscle group into the left and right side of the muscle group. A multi-stage training scheme has been used, wherein 20 datasets where initially labeled manually by L.K. (5 years of experience in musculoskeletal imaging). A first network, trained on the initial labels, was used to predict masks on 50 further datasets. In turn, all predictions were manually corrected and used for a subsequent training on the joint set of 70 annotated imaging volumes. All four contrasts (water, fat, in- and opposed-phase) were passed as input to the neural network. The final network performed predictions on the available 11026 datasets.

##### Mask Post-processing

To ensure robust feature generation, masks were post-processed (after their prediction) by a dedicated connected component labeling heuristic, which removes small isolated misclassifications automatically. Misclassifications have been defined as regions with a relative voxel amount of $< 2.5\%$ compared to the largest connected region. Larger but disjoint regions may indicate isolated but valid mask components that have to be visually inspected within a manual consistency check. In addition, a morphological erosion filter was applied to the masks prior to the texture feature extraction, to ensure that boundary regions of the muscles which may include partial tissue compartments of varying characteristics in intensity, texture or homogeneity, are not included in subsequent calculations.

##### Manual consistency check

**Fig. 2:**
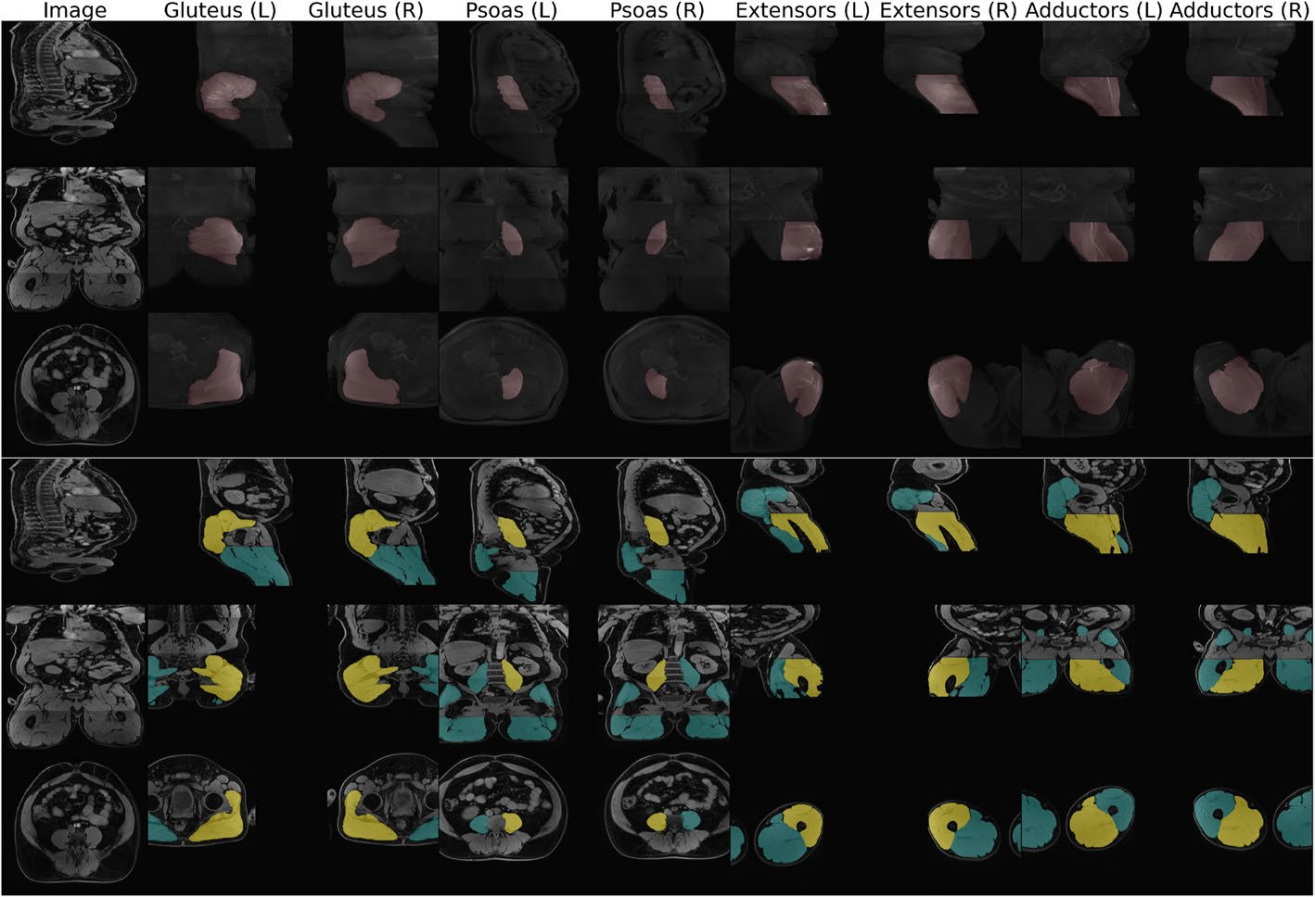
Overlay plot used for manual consistency check of predicted segmentation masks. All muscle groups for the left and right side are presented alongside the water contrast. The upper three rows show maximum intensity projections (MIP) along all three axes (sagittal, coronal, axial) with masked areas highlighted. In the lower three rows cross sections along all three axes are shown with yellow masks indicating the label of interest and turquoise masks representing all other present labels

A manual consistency check of all predicted segmentation masks alongside the imaging data was performed. This ensures no severely wrongful segmentation mask prediction was included in the feature extraction or corrupted imaging raw data was used. As such, an overlay plot of the predicted masks on the water image was generated for each subject (see Fig. 2). In this overview all four muscle groups (left and right) are illustrated along three orthogonal views (sagittal, coronal, axial). The content is depicted in two different ways: a maximum intensity projection (MIP) of the masked muscle regions (in the upper row) and cross sections along the geometric mean of the muscle volumes indicated in yellow (with other segmentation groups in turquoise) in the lower rows. This enables a simple visual inspection and identification of mask errors, the presence of fat-water swaps of the specific Dixon sequence or wrongful header (and thereby orientation) information. The visual check was performed based on the aforementioned plots by L.K.. Hereby, a total number of 344 subjects were discarded in the manual consistency check; 78 were excluded due to fat water swaps and 266 have been discarded due to erroneous segmentation mask predictions (including wrong mask orientations due to incorrect header information), leading to 10682 remaining subjects.

##### Texture Feature Extraction

In this work, the python package Pyradiomics [30] was employed to perform the extraction of various texture features. Pyradiomics provides a solid and well-tested basis of robustly implemented features for our subsequent selection. The tool was integrated in our radiomics pipeline (see Fig. 1). The segmentation masks generated in the preceeding step were used to select the water and fat contrasts of the two-point Dixon-weighted MR sequence. We extracted features for each muscle region for the water as well as fat image. Hereby first order statistics, gray level cooccurence matrix (glcm), gray level run length matrix (glrlm), gray level size zone matrix (glszm), neighboring gray tone difference matrix (ngtdm), gray level dependence matrix (gldm) were created. Different from studies as in [11], we also include common shape features (e.g. volume, area, sphericity, compactness, and elongation) alongside the texture features due to the availability of generated muscle masks. Border regions that may contain unwanted texture variations due to partial volume effects resulting in voxel bleeding or information from neighboring content, are excluded by a morphological erosion filter. Images were normalized prior to the feature extraction and bilinear resampled to a consistent resolution of $3\textrm{mm} \times 3\textrm{mm} \times 3\textrm{mm}$. Besides original features, variations of the image content by application of Laplacian of Gaussian (LoG) and Wavelet transformations were included, resulting in an overall number of 4060 available texture features per muscle group per side for each individual subject. In 10 subjects features couldn’t be generated due to an underlying corruption of the respective raw data. Leaving 10672 subjects for the subsequent feature selection process.

##### Auxiliary Targets

**Fig. 3:**
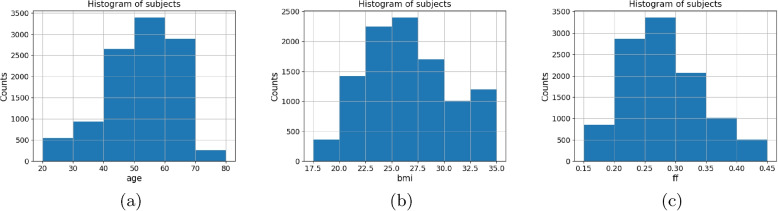
Histograms of investigated subgroups for individual targets **a** age, **b** body mass index (BMI), and **c** fat fraction (FF). Subjects exceeding the bounds are added to the first or last bin

Besides the feature extraction, we used image meta information to provide surrogate targets for the subsequent selection (in the absence of clinical outcome data). The auxiliary information includes the age, the BMI as well as the FF as indicators of the body type and composition. The age was extracted from available meta information, the BMI was calculated based on the height and weight in the meta information and the FF was calculated based on imaging information in combination with mask predictions. The FF was calculated for each individual muscle (per side) based on the water and fat image intensities of the selected sequence in the region of interest: $\textrm{FF} = \sum {i_{\textrm{fat}}}/{(i_{\textrm{water}} + i_{\textrm{fat}})}$ with intensity values *i* at the voxel in the relevant segmentation mask of each muscle. The mean ± standard deviation (median) was $51.85 \pm 11.40$ (53.00) years for age and $26.81 \pm 4.71$ (26.14) kg/m$^2$ for the BMI. The FF for each muscle group was $23.45 \pm 5.26$ $(23.08) \%$ for the psoas, $27.86 \pm 6.56$ $(27.04) \%$ for the gluteus, $12.11 \pm 3.30$ $(11.58) \%$ for the extensors and $19.94 \pm 5.13$ $(19.39) \%$ for the adductors. For the subgroups, we set bin widths of histograms as illustrated in Fig. 3. Each auxiliary target with values falling below or exceeding the shown range were added to the first or last bin respectively. The auxiliary targets are further explored in Subgroup differences section.

#### Feature selection

**Fig. 4:**
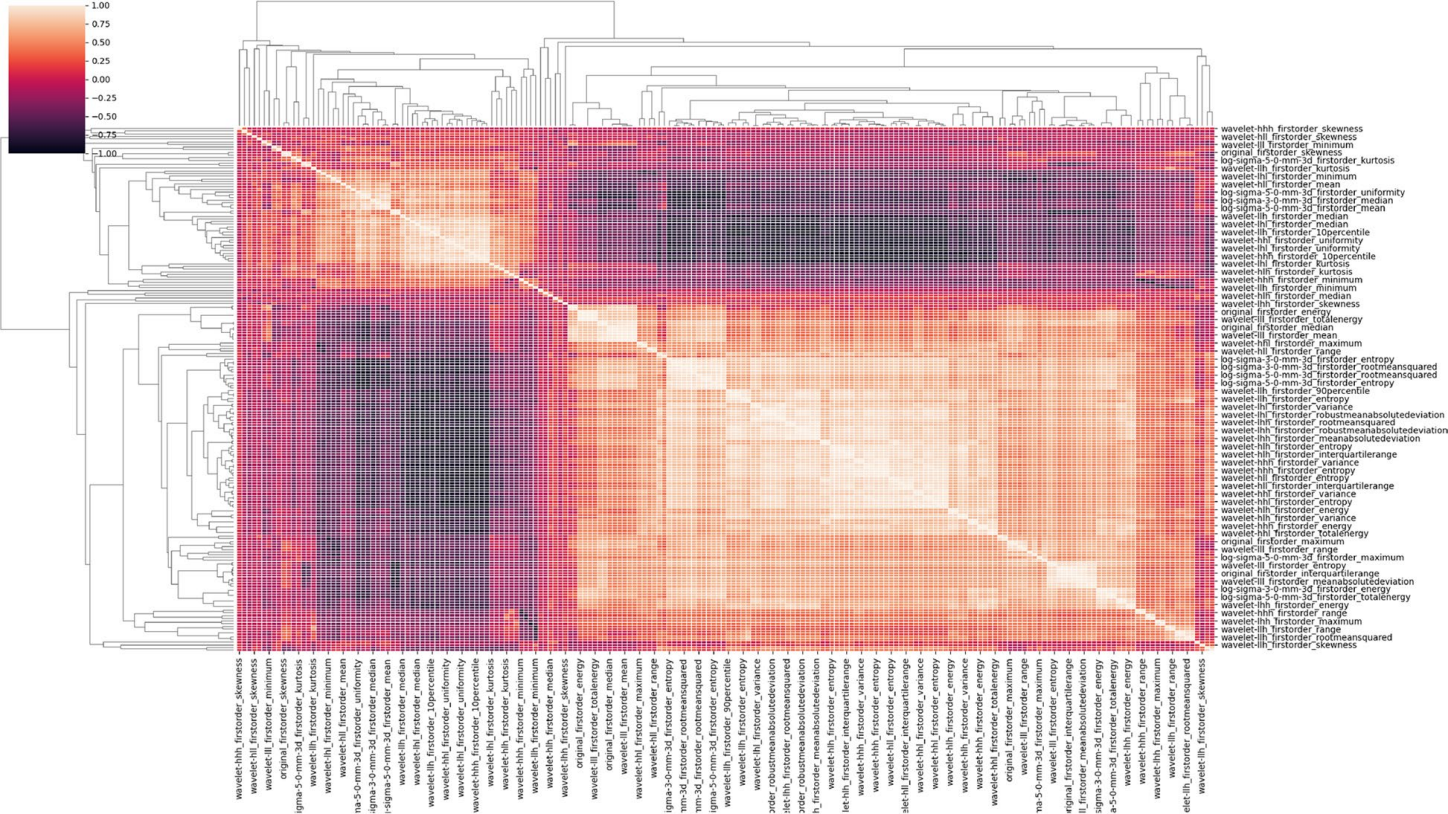
Exemplary intra-muscle feature correlations for extracted first-order radiomics features of the Dixon water contrast. High intra-muscle correlation values are present, indicating large collinearity between several features. A dendrogram on the left and top border shows a hierarchical clustering based on cluster distances to respective centroids

To identify relevant and meaningful texture features, several selection steps were implemented (see Fig. 1b), which ensured that selected features adhere to certain conditions. The conditions aimed to capture important correlations as well as differences between subjects or subgroups. In radiomics pipelines, texture features need not only to be extracted robustly, but also be selected in a consistent fashion. Thus, in *Step I* cluster-representatives for several highly correlated features were selected. This is required since the regions of interest of the predominantly healthy muscle tissue are more regular in texture compared to e.g. cancerous tissue for which radiomics features are employed usually. This resemblance between features is illustrated by the averaged intra-muscle correlations between first order statistic features of the water contrast in the color-coded Fig. 4. The illustrated correlations were averaged between both sexes and all muscles before the clustering, so that identical texture feature representatives are used across all investigated cases. In the figure, high intra-muscle correlation values were present, indicating large collinearity between several features. The dendrogram on the edges of the correlation matrix shows a hierarchical clustering based on the distance to respective cluster centroids. Distances below a threshold $< 0.1$ lead to an inclusion in the respective cluster and to an exclusion for values above. Representatives were taken in alphabetical order with priority given to original (unchanged) features over transformed LoG features and then wavelet feature variants. The clustering was further limited to features of the same sub-category (e.g. first order, glcm, glrlm, and more) to keep a rich set of variations.

Subsequent steps of the feature selection (Fig. 1b), II - V) were performed in a cross-validation strategy with five disjoint folds of subjects. This scheme was employed since auxiliary information was used as selection criteria which can lead to different outcomes for subject-specific data characteristics. Hereby, four folds were considered as the training data and the remaining fold data was considered as the validation data. This process was repeated for each possible validation fold. For the given datasets, the training data contained 8536 subjects and the validation data contained 2134 subjects.

In *Step II*, features with a weak mean intra-feature correlation $< 0.7$ between the left and the right masked region in a muscle of a training subject were discarded. The reasoning behind this selection was that a low intra-feature correlation value indicates poor reproducibility e.g. due to the susceptibility to noise, texture inhomogeneieties or the impact of present imaging artifacts.

In *Step III*, the amount of features was further reduced by a variance inflation factor (VIF) calculation [38]. The VIF is a ratio of variances (related to the coefficient of determination $R^2$) that provides a measure for the linear relationship between multiple parameters (multi-collinearity). Based on a leave-one-out linear regression, features with the highest inflation factor were removed iteratively if the VIF was above $>10$ which is commonly regarded as indicating high multi-collinearity [38]. Ignoring the remaining multi-collinearity can lead to adverse effects in epidemiological studies [39]. Hence, this step is intended to reduce the remaining collinearity which may otherwise negatively impact subsequent importance calculations and thereby the selection of texture features with meaningful inter-subject variability. To keep a diverse set of features, the variance inflation calculation was again performed for each feature sub-category, such as first order or glcm, separately.

In *Step IV*, a Boruta feature selection heuristic [40] was employed. This method has been shown to be an excellent choice for the selection of omics data in combination with random forests [41]. It estimates the feature importance with respect to the employed auxiliary information (age, BMI, FF). Hereby, a random regression forest was applied and trained, in which random permutations of features are compared with true extracted features. The random forest contained 100 trees with a squared error split criterion and a maximum tree depth of 5. A heuristic was used in which extracted features are considered eligible only if they are more discriminative than the artificially generated alterations. Otherwise, the respective features were discarded. The selected features vary based on the chosen auxiliary target.

In *Step V*, a final feature importance for the post-selection ranking was calculated of the remaining features. Again, a random regression forest was used to calculate a permutation importance [42]. In this case, a similar random forest, with 100 trees, a squared error split criterion, and a maximum tree depth of 8, was used. We note, that the post-selection importance was identified on the validation data of the respective fold instead of the training data (as done in prior selection steps). This importance was in turn used for the following ranking scheme to identify overall meaningful features and their order. For a respective fold the proposed ranking scheme follows the formula

$$\text{rank}_{\text{fold}_{i}, \text{feature}_{j}} = \frac{1}{N_{\text{repeats}} \cdot N_{\text{targets}}} \sum\limits _{k}^{N_{\text{repeats}}} \sum\limits _{l}^{N_{\text{targets}}} \frac{\text{rank}_{i, j, k, l}}{|N_{{\text{features}, i, l}} |}$$

with $N_{\textrm{repeats}}$ describing the number of random permutations to determine the feature importance, $N_{\textrm{features}}$ depicting the number of selected features in *Step IV*, and $N_{\textrm{targets}}$ describing the number of auxiliary information targets (if the ranking is averaged across all targets). $\textrm{rank}_{i,j,k,l}$ represents the rank of the relative post-selection importance of feature *j* for the given target *k* and fold *i*. The rank is normalized between 0 (first) and 1 (last). Features that were not selected in the selection process for one of the auxiliary targets but were present for at least one target, were ranked last, i.e., with a value of 1.

### Statistical evaluation

The post-selection ranking importance is given as mean ± standard deviation of all folds. To confirm the statistical significance of differences between subgroups, non-parametric analyses of variance were performed due to the non-normal distribution of range bound extracted features. Thus, a Kruskal-Wallis H-test is applied to identify significant differences between the subgroups. This test provides a one-way analysis of variance (ANOVA) on ranks and determines if samples from the different subgroups originate from the same distribution. We apply a Bonferroni correction, due to the concurrent investigation of the first ten features in the following, leading to a significant *p*-value threshold of 0.005. In addition, we performed a Mann-Whitney U test, for differences between each respective male and female subgroup (e.g. age 30-39 male vs 30-39 female). It is another test on ranks that indicates if two independent groups are significantly different from each other. Depending on the number of subgroups a Bonferroni corrected *p*-value was used (0.008 for six subgroups, 0.007 for seven subgroups). In addition, to give an estimate of the multi-class classification performance based on the selected features, we trained a classification random forest with 500 trees, an entropy split criterion, and a maximum tree depth of 10. Different from the post-selection importance, this tree was trained on the training data (four folds) and applied to the left-out (validation) fold with respect to the introduced auxiliary target subgroups.

## Results

### Feature selection

Figure 1 indicates the amount of unique features that remained after each selection step for features from the generation pipeline for a joint selection on all available muscle groups. The values depicted show mean [lower bound, upper bound] of features selected across all folds. 524 out of the 1015 unique features from the water as well as the fat contrast remained after the cluster-representative selection (*Step I*). For the cross-validated steps (*Step II-IV*) 306 [302, 309] features remained after the intra-class correlation calculation (*Step II*) and 70.2 [68, 72] features were available after the VIF selection (*Step III*). For the Boruta heuristic (*Step IV*) 65.2 [62, 68] features for age, 53.0 [47, 59] features for BMI, and 36.4 [33, 39] features for FF were deemed eligible. We also report the amount of selected features for separate modalities (with shape features included in both cases) by mean [lower bound, upper bound] (unique features across all folds). For the water contrast 34.6 [32, 37] (45) features for age, 31.0 [29, 36] (40) features for BMI, and 13.8 [12, 16] (18) features for FF were present for the subsequent post-selection calculation. For the fat contrast 44.2 [41, 47] (58) features for age, 30.6 [21, 40] (52) for BMI, and 25.2 [21, 28] (36) features for FF remained. Further amounts for individual muscles are given in Table 1. In most cases, the number of unique features seen across all folds was higher for the features of the fat contrast than for the water contrast. We note that minor variations, such as different wavelet filter combinations, can inflate the amount of unique features.

**Table 1:** Varying amounts of selected texture features across all folds for the water and fat contrast and different auxiliary targets. Amounts are given by mean [lowest amount, highest amount] (amount of unique features)

contrast	muscle	age	BMI	FF
water	all	34.6 [32, 37] (45)	31.0 [29, 36] (40)	13.8 [12, 16] (18)
	gluteus	37.6 [35, 39] (49)	37.8 [37, 39] (50)	24.2 [21, 27] (38)
	psoas	26.4 [25, 28] (35)	24.8 [22, 26] (34)	21.2 [19, 25] (34)
	extensors	33.6 [29, 35] (48)	28.6 [26, 33] (40)	31.6 [28, 37] (48)
	adductors	39.6 [33, 44] (61)	23.0 [20, 28] (48)	33.8 [28, 42] (61)
fat	all	44.2 [41, 47] (58)	30.6 [21, 40] (52)	25.2 [21, 28] (36)
	gluteus	45.4 [42, 48] (60)	45.6 [41, 49] (58)	39.4 [35, 43] (56)
	psoas	32.0 [31, 33] (39)	31.6 [30, 33] (39)	22.6 [19, 25] (27)
	extensors	31.4 [29, 33] (51)	30.4 [25, 34] (52)	42.6 [38, 51] (70)
	adductors	34.6 [32, 37] (60)	37.0 [30, 41] (64)	36.0 [29, 41] (57)

### Feature importance ranking

**Fig. 5:**
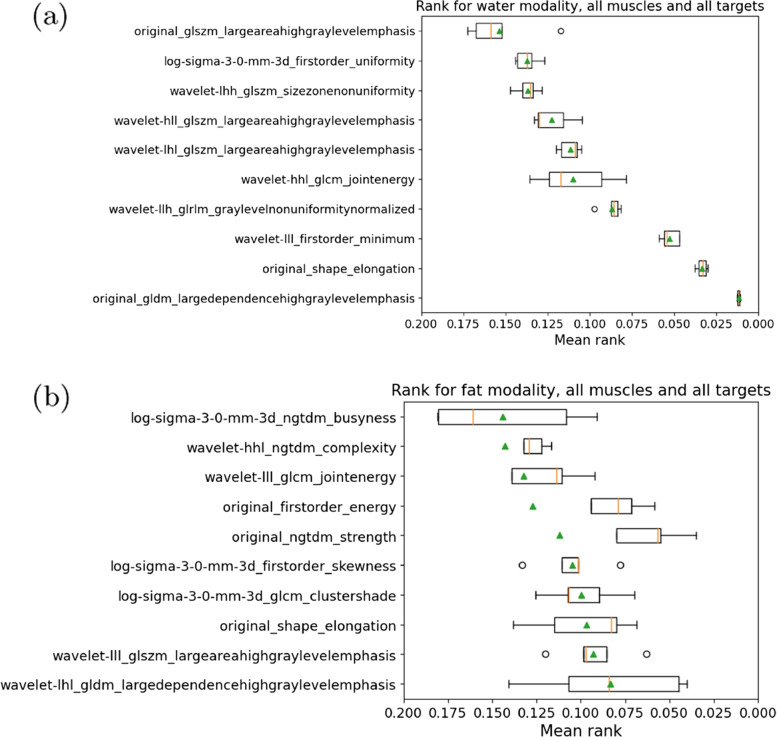
Boxplots of rankings based on permutation importance across targets with values between 0 (first) and 1 (last) for all targets combined in **a** water and **b** fat contrast

Based on the selected features, we calculated the importance ranking across all folds. Exemplary rankings thereof averaged across all targets for the first ten features of the water as well as the fat contrast are visualized via boxplots in Fig. 5. Green triangles indicate the mean rank and red lines the median value. Large differences between mean and median values indicate different ranks for different targets and can further vary due to separate ranking results for each fold. For the water contrast, the mean ranking showed a clear distinction between the ranking score of certain features including the top performing features. We see a plethora of different features from different subgroups such as first order, shape, gldm, glrlm and their wavelet and log-sigma variants scoring low overall ranks. For example, for the second water contrast features, we see a shape feature, namely the original_shape_elongation which is highly dependent on the muscle shape and can thus vary greatly for different muscles. Considering all muscles, it scores nonetheless with a low rank. For the fat contrast the ranking becomes noisier indicated by larger bounding boxes and in some cases strong differences between the mean and median rank as seen e.g. for original_ngtdm_strength and original_firstorder_energy.

**Fig. 6:**
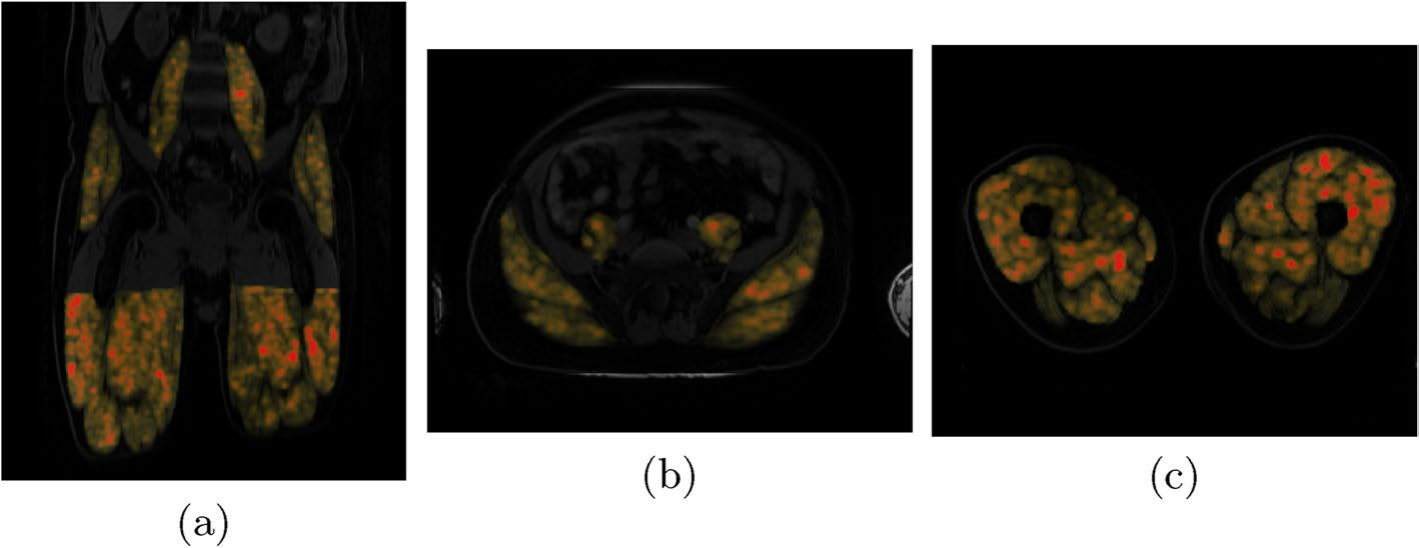
Exemplary visualization of voxel-wise texture feature original_gldm_largedependencehighgraylevelemphasis in one subject for **a** a coronal view in all muscle groups, **b** axial views in gluteus and psoas, and **c** axial views through adductors and extensors

For illustration, we added a voxel-wise representation of the first ranking feature original_gldm_largedependencehighgraylevelemphasis for all investigated muscles across a coronal and two axial views in Fig. 6. We note, that in contrast to this visualization, the processed texture features used for the ranking are based on mean values, which are aggregated across respective masked regions for each muscle.

**Fig. 7:**
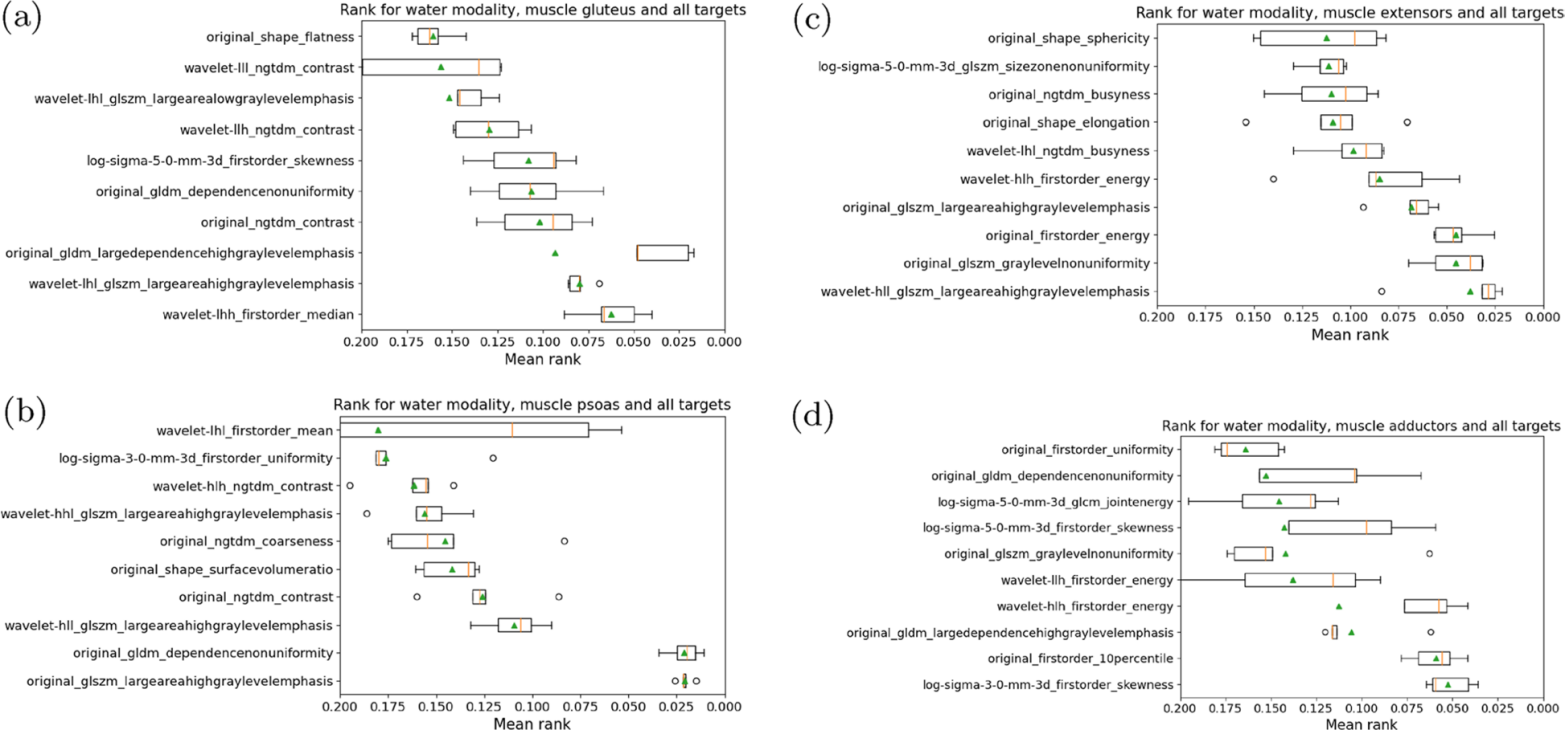
Boxplots of feature rankings based on permutation importance in water contrast images for **a** glutues, **b** psoas, **c** extensors and **d** adductors

We report the ranking for individual muscles of the water contrast averaged on all three targets in Fig. 7. Hereby variants (original, LoG, wavelet) of the gldm_largedependencehighgraylevelemphasis and glszm_largeareahighgraylevelemphasis scored low on all four muscle groups, indicating that these features are important to identify at least one of the auxiliary targets (age, BMI or the FF). The shape elongation is less important for each individual muscle, as it does not aid in differentiating characteristics within the same muscle group compared to the presence of multiple muscles (thereby indirectly allowing for a better estimation of the FF). Again, we see the original as well as LoG and wavelet features across varying different subgroups. The gluteus, psoas and the extensors showed tight boxplots with close mean and median values leading to robust rankings. The ranking for the adductors showed two good performing features for the lowest ranks and higher variability for subsequent features. We note, that this muscle group was partially cut off in some cases (due to the FOV placement of the imaging data), which may lead to lower prediction accuracy and thereby higher mask coverage errors.

**Fig. 8:**
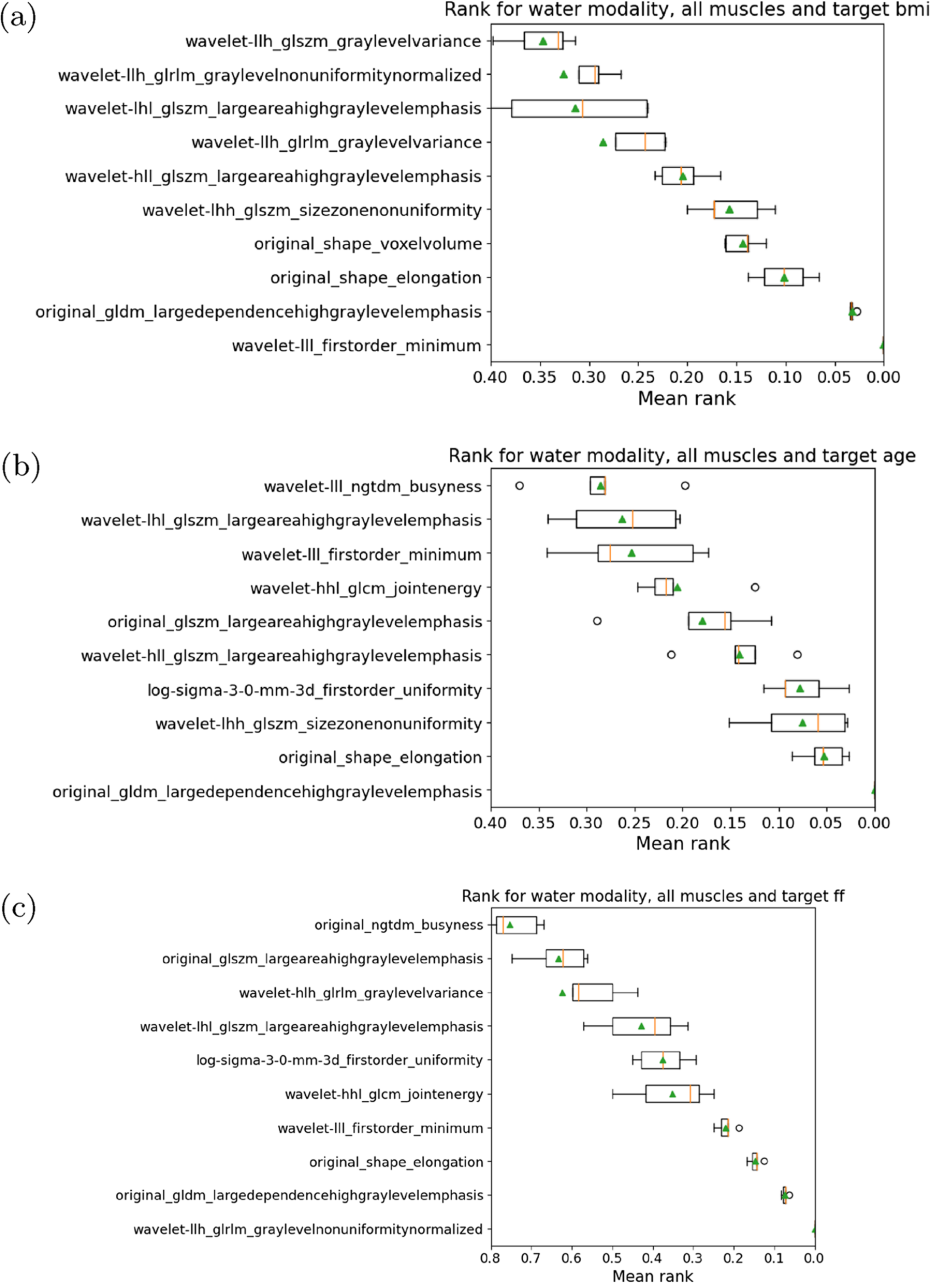
Boxplots of feature rankings based on permutation importance in contrast water for individual targets a) age, b) body mass index (BMI), and c) fat fraction (FF)

To further illustrate the resulting ranking, we depict the ranking averaged across all muscles for individual targets by means of boxplots in Fig. 8. In all cases a well-structured ranking with distinctive relative feature importances was produced. Across all three targets, the ranking varied drastically with an almost linear trend of the mean rank in all three cases compared to overall rankings. There was also a higher difference in the mean rank between the first and tenth feature compared to the overall rankings as well as the averaged rankings per muscle. Also different from the previous examples, for each auxiliary target there is a texture feature which achieves a mean rank $< 0.01$. This indicates that one feature scores best very consistently. For the age target the gldm_largedependencehighgraylevelemphasis feature remains the most important feature. For BMI a wavelet variant of the firstorder_minimum and for FF a wavelet variant of the glrlm_graylevelnonuniformitynormalized achieved the lowest rank.

### Subgroup differences

**Fig. 9:**
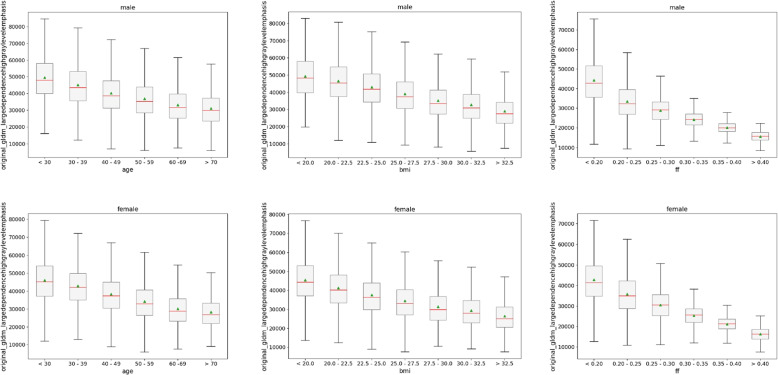
Texture feature values on water contrast of lowest ranked feature original_gldm_largedependencehighgraylevelemphasis for individual targets age (left column), body mass index (BMI) (middle column) and fat fraction (FF) (right column) separated by sex (top row: male, bottom row: female)

**Fig. 10:**
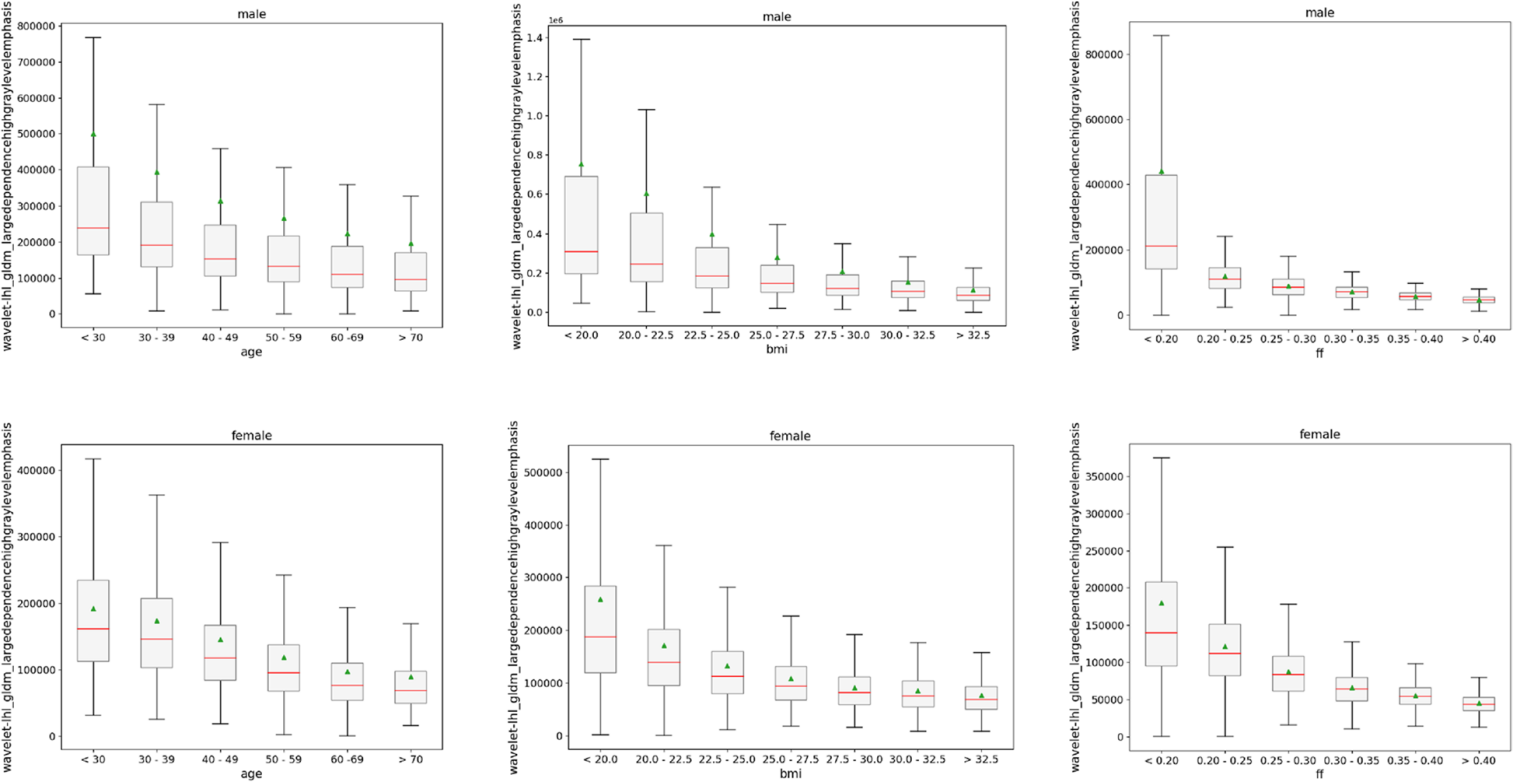
Texture feature values on fat contrast of lowest ranked feature wavelet-lhl_gldm_largedependencehighgraylevelemphasis for individual targets age (left column), body mass index (BMI) (middle column) and fat fraction (FF) (right column) separated by sex (top row: male, bottom row: female)

**Fig. 11:**
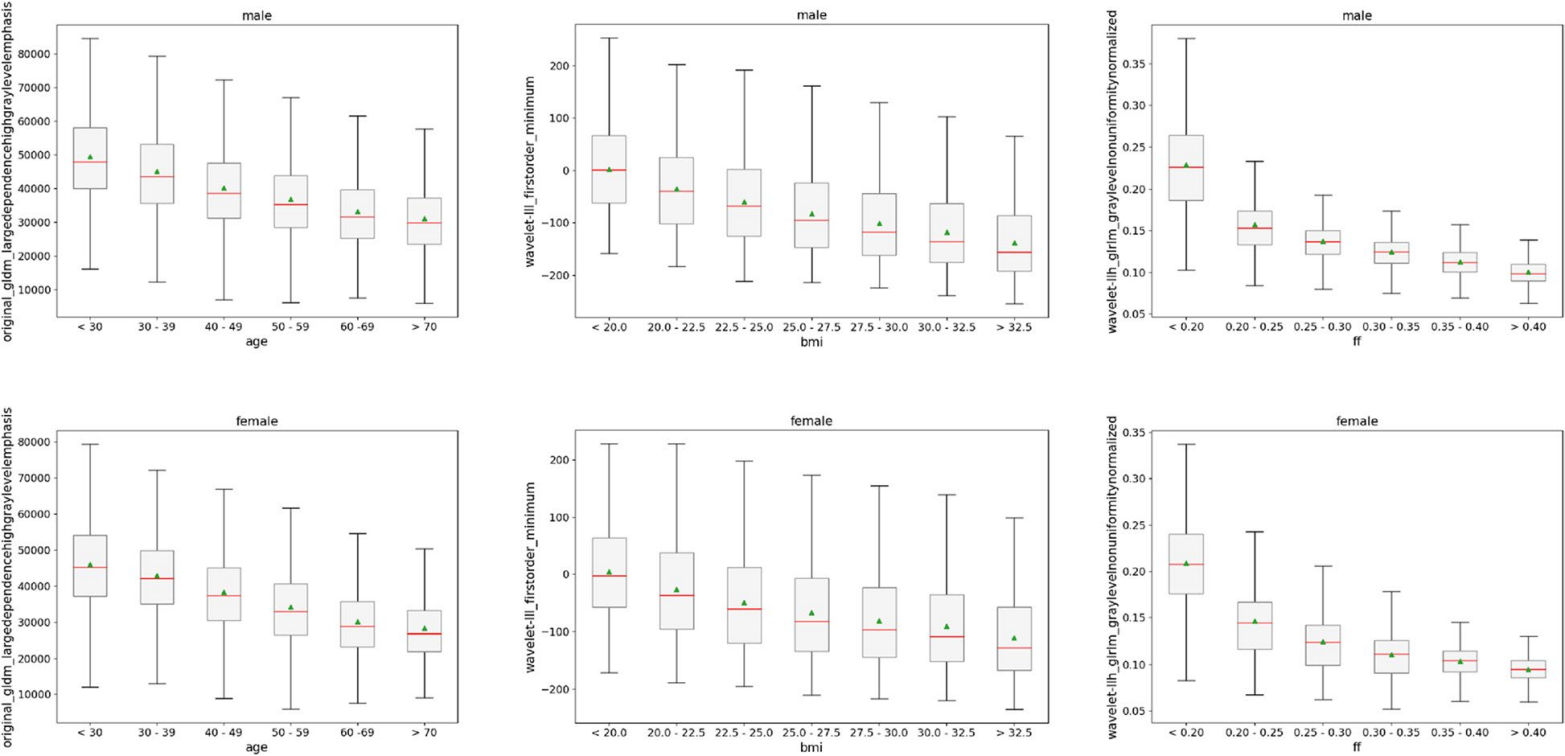
Lowest ranked texture feature values on water contrast based on individual targets; original_gldm_largedependencehighgraylevelemphasis for age (left column), wavelet-lll_firstorder_minimum for body mass index (BMI) (middle column) and wavelet-llh_glrlm_graylevelnonuniformitynormalized for fat fraction (FF) (right column) in male (top row) and female (bottom row) subjects

**Fig. 12:**
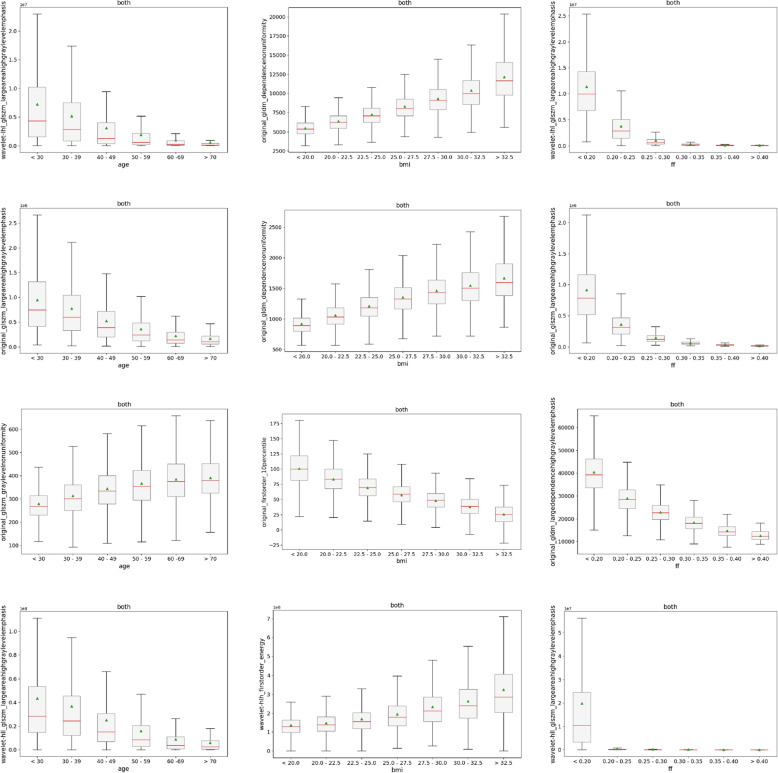
Lowest ranked texture feature values on water contrast for each muscle based on an individual targets age (left column), body mass index (BMI) (middle column) and fat fraction (FF) (right column) in both male and female subjects combined; first row gluteus, second row psoas, third row adductors, fourth row extensors

With the ranking based on the feature importance of selected features, we can illustrate subgroup differences of the top performing features. We depict the distribution of some exemplary texture feature values for male and female subgroups below. Hereby, boxplots separated by males and females for each auxiliary targets are presented based on the feature values for the lowest ranked and thereby best performing features included in Feature importance ranking section. These include the best feature of the ranking averaged across all muscles and all targets on the water contrast (Fig. 9), as well as the feature of the ranking averaged across all muscles and all targets on the fat contrast (Fig. 10), the features of the ranking for each target averaged across all muscles on the water contrast (Fig. 11), and the features for the ranking of each target and each muscle on the water contrast (Fig. 12). To get a broader picture of the top performing features, further quantitative results of the Kruskal-Wallis H-test between subgroups of the ten lowest ranking features and *p*-values of the Mann-Whitney U-test between respective male and female subgroup bins are reported in Tables 2 and 3. Hereby features, according to the ranking identified for all targets as well as individual targets across all muscles have been considered. The figures and respective table entries are explored in the subsequent paragraphs.

**Table 2:** Highest *p*-values of the Kruskal-Wallis H-test between subgroups of the ten first ranked features. Lowest *p*-values are consistently $< 0.001$

contrast	target	*p*-value					
		age		BMI		FF	
		male	female	male	female	male	female
water	all	$< 0.001$	0.034	$< 0.001$	$< 0.001$	$< 0.001$	$< 0.001$
	individual	$< 0.001$	0.034	$< 0.001$	$< 0.001$	$< 0.001$	$< 0.001$
fat	all	$< 0.001$	0.034	$< 0.001$	$< 0.001$	$< 0.001$	$< 0.001$
	individual	$< 0.001$	0.034	$< 0.001$	$< 0.001$	$< 0.001$	$< 0.001$

In Fig. 9, we see the first ranking feature gldm_largedependencehighgraylevelemphasis across all muscles and all targets on the water contrast for male and female subgroups. The main body of the boxes indicates the first (lower bound) and third quartile (upper bound). Values of neighboring subgroups can overlap substantially. However, the mean (green triangles) and median (red lines) show monotonic trends of its values. These trends are present for male and female subjects as well as for different targets. The Kruskal-Wallis *p*-value for the original_gldm_largedependencehighgraylevelemphasis feature consistently showed a *p*-value < 0.001 indicating statistically significant differences between subgroups for all available auxiliary targets. In addition, all of the lowest ranked ten features of the water contrast identified in the selection pipeline including the final ranking, exceeded a *p*-value of 0.005 for males (see Table 2). For females, only one of the ten features (original_shape_elongation) did not show significant changes between age subgroups with a maximum *p*-value of 0.034 (being above the corrected threshold of 0.005). Note that all other nine texture features remained below the significance threshold of 0.005 for females. For the Mann-Whitney-U test, most subgroup comparisons showed a *p*-value < 0.001, showing significant differences between male and female texture feature values for features identified for the water contrast and all targets. Prominently, there are exceptions, where the *p*-value exceeds the significance thresholds. These outliers are especially for the first and last subgroup bins (where subjects in the long tails of the distributions are aggregated) with highest *p*-values of 0.059 for age, 0.465 for BMI and 0.003 for the FF. Respective features (indicated by the their rank from 1 to 10) and the number of the histogram bins associated with the highest *p*-value are reported in Table 3.

**Table 3:** Highest *p*-values of the Mann-Whitney U-test between male and female subjects of identical subgroups of the ten first ranked features. Lowest *p*-values are consistently $< 0.001$

contrast	target	age			BMI			FF		
		*p*-value	feature	bin	*p*-value	feature	bin	*p*-value	feature	bin
water	all	0.059	8	1	0.465	3	1	0.003	1	6
	individual	0.059	3	1	0.465	1	1	0.003	2	6
fat	all	0.380	6	6	0.498	8	1	0.440	4	6
	individual	0.380	10	6	0.470	4	6	0.440	2	6

In Fig. 10 we see the respective texture feature values for the wavelet-lhl variant of the gldm_largedependencehighgraylevelemphasis feature on the fat contrast. It follows similar trends as the first ranked variant of the water contrast, yet the decrease for all targets across bins is less linear. Notably, there is a large difference between median and mean values for males indicating a distribution skewed to higher values. This is also present, albeit in a less prominent fashion, for females. Nonetheless, clear differences between each subgroup can be seen again with a Kruskal-Wallis *p*-value < 0.001 for the shown feature values for males and females alike. As illustrated in Table 2, this held also true for the ten lowest ranked texture features of the fat contrast for the BMI and FF target. For age, the *p*-value is again 0.034 since the shape_elongation feature is contrast independent and was thereby also present in the ranking for the fat contrast. *p*-values of the lowest ten ranked features in the Mann-Whitney U test between male and female subgroups are higher compared to the water contrast, with values of 0.380 for age, 0.498 for BMI and 0.440 for FF exceeding the significance thresholds of 0.008, 0.007, and 0.008 respectively.

In Fig. 11 we show the first ranked feature for individual targets; for age the feature remains gldm_largedependencehighgraylevelemphasis, for the BMI we depict the wavelet-lll variant of the firstorder_minimum and for the fat fraction we show the wavelet-llh variant of the glrlm_graylevelnonuniformitynormalized. In all cases, the features show similar trends independent of their vastly different range of values. For BMI, the means decreases for male and female subjects alike. All ten lowest ranked texture features remained below a *p*-value of 0.001 for the Kruskal-Wallis test. For BMI, the significant *p*-value of 0.007 for the Mann-Whitney U test was exceeded for the first subgroup ($p= 0.465$). The other subgroups all showed significant differences between male and female subjects. Highest *p*-values are again noted in Table 2 and 3 for both contrasts.

Lastly, in Fig. 12 we illustrate boxplots for all individual muscle groups and their respective first ranked feature (for each individual target and joint male and female subjects) in each row. The columns again include the different auxiliary targets. Subgroup differences varied more compared to the aforementioned boxplots with monotonic decreases as well as increases of the mean and median feature values. Scales of the subgroups can also vary greatly, as can be seen for the FF of the extensors. Three variants of the largeareahighgraylevelemphasis were present (original for the psoas, wavelet variants for the gluteus and the extensors) indicating the robustness and sensitivity of this feature with respect to the auxiliary targets. In addition, first-order features such as energy and the 10th percentile were able to distinguish well between subgroups of the BMI target.

### Prediction performance on subgroups

**Fig. 13:**
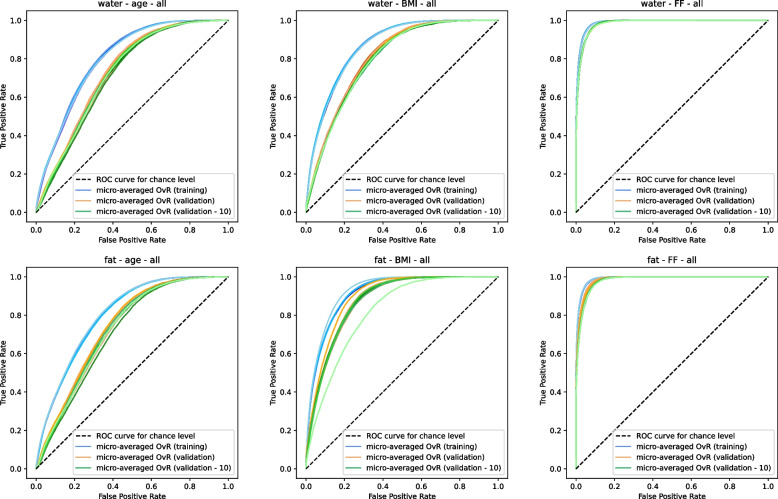
Receiver operator characteristic (ROC) curves for micro-aggregated one-vs-rest (OvR) multi-class classification on all muscle groups for the defined auxiliary target subgroups. Curves are illustrated for the training folds in blue (training) and the respective remaining validation folds in orange (validation) as well as for the ten lowest ranked texture features on the validation folds in green (validation - 10)

**Fig. 14:**
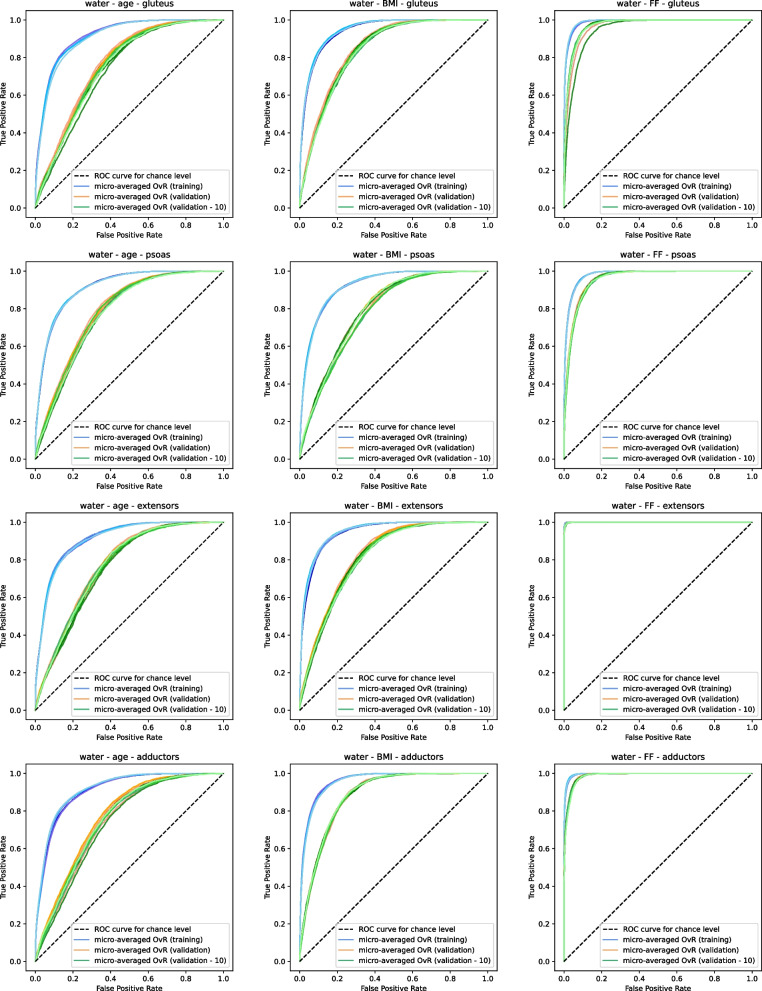
Receiver operator characteristic (ROC) curves for micro-aggregated one-vs-rest (OvR) multi-class classification on the water contrast for individual muscle groups for the defined auxiliary target subgroups. Curves are illustrated for the training folds in blue (training) and the respective remaining validation folds in orange (validation) as well as for the ten lowest ranked texture features on the validation folds in green (validation - 10)

To evaluate the performance of selected features as well as the proposed ranking scheme, we calculated receiver operator charactersitic (ROC) curves and their derived area under the curve (AUC) scores. The ROC curve is a means to illustrate the ratio between true and false positive rates for a binary classifier. It depicts lines that indicate the ability to detect true positives when varying the threshold of false positives. The AUC is the area under the respective line and provides a scalar value for its overall performance. We employ it for the multi-class classification performance on the introduced age, BMI, and FF subgroups. Hereby, the classification random forest predictions are considered in a One-vs-Rest (OvR) setting (where one class is considered the positive and the remaining classes as negatives) and averaged to calculate ROC curves. Exemplary curves for the joint selection on all muscles for both contrasts are illustrated in Fig. 13. Based on a feature selection with individual muscles, Fig. 14 shows prediction curves for all auxiliary targets for the water contrast. In both figures, the training as well as validation performance are depicted by a separate line for each fold. The figures also include a further selection of the 10 lowest ranked features (validation - 10) based on the calculated post-selection importance. The corresponding AUC values for all depicted curves, as well as further cases based on the fat contrast, are reported in Table 4. In addition, we provide mean accuracy values for the correct prediction of the specific subgroup in Table 5.

**Table 4:** Area under the curve (AUC) of the receiver operator characteristic (ROC) curves for micro-aggregated one-vs-rest (OvR) multi-class classification of the defined auxiliary target subgroups. Performance metric was evaluated on the validation sets of each respective fold. Values are given by mean ± standard deviation across all folds for training (train), validation (val) as well as for the top 10 ranking features according to the respective ranking scores (train 10, val 10)

contrast	muscle	train	train 10	val	val 10
		age			
water	all	$80.30\pm 0.33\%$	$78.53\pm 0.14\%$	$73.03\pm 0.70\%$	$72.24\pm 0.55\%$
	gluteus	$91.41\pm 0.34\%$	$88.37\pm 0.21\%$	$76.43\pm 0.50\%$	$74.90\pm 0.81\%$
	psoas	$91.46\pm 0.09\%$	$89.29\pm 0.28\%$	$78.54\pm 0.67\%$	$77.60\pm 0.53\%$
	extensors	$91.10\pm 0.28\%$	$89.02\pm 0.10\%$	$76.94\pm 0.73\%$	$76.34\pm 0.65\%$
	adductors	$91.44\pm 0.36\%$	$88.58\pm 0.28\%$	$76.09\pm 0.94\%$	$74.93\pm 0.88\%$
fat	all	$80.45\pm 0.20\%$	$78.06\pm 0.17\%$	$73.63\pm 0.70\%$	$72.32\pm 0.74\%$
	gluteus	$91.50\pm 0.36\%$	$88.84\pm 0.27\%$	$76.70\pm 0.58\%$	$75.79\pm 0.62\%$
	psoas	$91.45\pm 0.16\%$	$89.57\pm 0.16\%$	$79.79\pm 0.47\%$	$79.37\pm 0.45\%$
	extensors	$91.04\pm 0.10\%$	$89.73\pm 0.16\%$	$78.49\pm 0.63\%$	$78.17\pm 0.82\%$
	adductors	$91.17\pm 0.16\%$	$87.79\pm 0.22\%$	$76.47\pm 0.98\%$	$74.82\pm 0.78\%$
		BMI			
water	all	$86.80\pm 0.21\%$	$85.05\pm 0.11\%$	$80.68\pm 0.30\%$	$79.87\pm 0.09\%$
	gluteus	$94.54\pm 0.21\%$	$92.72\pm 0.14\%$	$84.63\pm 0.46\%$	$93.92\pm 0.31\%$
	psoas	$92.47\pm 0.09\%$	$91.17\pm 0.21\%$	$79.14\pm 0.66\%$	$78.99\pm 0.73\%$
	extensors	$94.78\pm 0.26\%$	$93.00\pm 0.06\%$	$82.26\pm 0.43\%$	$81.39\pm 0.41\%$
	adductors	$95.91\pm 0.18\%$	$95.10\pm 0.22\%$	$88.31\pm 0.28\%$	$88.37\pm 0.18\%$
fat	all	$91.89\pm 0.46\%$	$90.02\pm 2.02\%$	$88.03\pm 0.89\%$	$86.42\pm 2.32\%$
	gluteus	$96.78\pm 0.05\%$	$95.97\pm 0.03\%$	$90.13\pm 0.37\%$	$90.48\pm 0.31\%$
	psoas	$96.49\pm 0.05\%$	$95.83\pm 0.06\%$	$88.84\pm 0.28\%$	$89.93\pm 0.18\%$
	extensors	$97.12\pm 0.08\%$	$96.37\pm 0.07\%$	$91.00\pm 0.50\%$	$91.34\pm 0.35\%$
	adductors	$96.66\pm 0.38\%$	$94.97\pm 0.29\%$	$89.38\pm 1.05\%$	$88.37\pm 0.59\%$
		FF			
water	all	$98.91\pm 0.03\%$	$98.90\pm 0.00\%$	$98.36\pm 0.03\%$	$98.36\pm 0.04\%$
	gluteus	$99.07\pm 0.07\%$	$98.61\pm 0.48\%$	$97.12\pm 0.33\%$	$96.80\pm 0.95\%$
	psoas	$98.18\pm 0.05\%$	$97.85\pm 0.03\%$	$95.83\pm 0.14\%$	$95.66\pm 0.13\%$
	extensors	$99.99\pm 0.00\%$	$99.98\pm 0.00\%$	$99.96\pm 0.00\%$	$99.96\pm 0.00\%$
	adductors	$99.65\pm 0.04\%$	$99.47\pm 0.02\%$	$98.86\pm 0.05\%$	$98.77\pm 0.08\%$
fat	all	$99.13\pm 0.02\%$	$98.73\pm 0.03\%$	$98.52\pm 0.09\%$	$98.11\pm 0.05\%$
	gluteus	$99.74\pm 0.02\%$	$99.35\pm 0.02\%$	$98.40\pm 0.05\%$	$97.95\pm 0.06\%$
	psoas	$99.18\pm 0.02\%$	$99.06\pm 0.04\%$	$97.62\pm 0.07\%$	$97.70\pm 0.06\%$
	extensors	$99.99\pm 0.00\%$	$99.98\pm 0.00\%$	$99.97\pm 0.00\%$	$99.96\pm 0.00\%$
	adductors	$99.62\pm 0.04\%$	$99.38\pm 0.01\%$	$98.82\pm 0.08\%$	$98.56\pm 0.11\%$

**Table 5:** Mean multi-class classification accuracy on the defined auxiliary target subgroups. Performance metric was evaluated on the validation sets of each respective fold. Values are given by mean ± standard deviation across all folds for training (train), validation (val) as well as for the top 10 ranking features according to the respective ranking scores (train 10, val 10)

contrast	muscle	train	train 10	val	val 10
		age			
water	all	$46.69\pm 0.83\%$	$42.71\pm 0.43\%$	$30.61\pm 1.05\%$	$29.11\pm 1.01\%$
	gluteus	$68.99\pm 1.09\%$	$62.34\pm 1.17\%$	$35.01\pm 079\%$	$32.78\pm 1.09\%$
	psoas	$68.28\pm 0.17\%$	$62.85\pm 0.44\%$	$38.59\pm 1.43\%$	$37.01\pm 1.47\%$
	extensors	$68.40\pm 0.49\%$	$63.20\pm 0.43\%$	$36.01\pm 0.88\%$	$34.90\pm 0.97\%$
	adductors	$68.33\pm 0.79\%$	$62.03\pm 0.72\%$	$35.08\pm 1.32\%$	$34.04\pm 1.01\%$
fat	all	$45.49\pm 0.67\%$	$39.94\pm 0.68\%$	$30.30\pm 0.81\%$	$27.73\pm 0.96\%$
	gluteus	$68.37\pm 0.56\%$	$61.69\pm 0.45\%$	$34.71\pm 1.00\%$	$34.12\pm 0.93\%$
	psoas	$67.09\pm 0.63\%$	$61.58\pm 0.72\%$	$39.40\pm 1.33\%$	$38.30\pm 1.48\%$
	extensors	$66.71\pm 0.19\%$	$63.43\pm 0.19\%$	$37.28\pm 1.04\%$	$37.17\pm 1.30\%$
	adductors	$67.14\pm 0.63\%$	$59.29\pm 0.78\%$	$35.02\pm 1.20\%$	$33.08\pm 0.96\%$
		BMI			
water	all	$53.15\pm 0.62\%$	$47.88\pm 0.35\%$	$35.92\pm 0.40\%$	$34.96\pm 0.37\%$
	gluteus	$72.63\pm 0.54\%$	$65.84\pm 0.37\%$	$42.87\pm 0.97\%$	$41.40\pm 0.32\%$
	psoas	$69.68\pm 0.36\%$	$65.60\pm 0.84\%$	$34.37\pm 1.00\%$	$34.37\pm 1.00\%$
	extensors	$75.55\pm 0.80\%$	$69.31\pm 0.34\%$	$38.35\pm 0.99\%$	$37.27\pm 0.92\%$
	adductors	$75.36\pm 0.88\%$	$71.13\pm 0.89\%$	$48.97\pm 0.94\%$	$48.84\pm 0.81\%$
fat	all	$61.90\pm 0.81\%$	$56.18\pm 3.75\%$	$48.96\pm 2.26\%$	$45.81\pm 3.66\%$
	gluteus	$77.09\pm 0.21\%$	$72.37\pm 0.21\%$	$53.02\pm 1.32\%$	$53.75\pm 1.01\%$
	psoas	$78.01\pm 0.30\%$	$72.47\pm 0.14\%$	$50.77\pm 0.91\%$	$52.68\pm 0.65\%$
	extensors	$78.88\pm 0.67\%$	$73.59\pm 0.26\%$	$55.17\pm 1.55\%$	$56.02\pm 1.59\%$
	adductors	$77.53\pm 1.39\%$	$69.88\pm 1.04\%$	$50.81\pm 2.76\%$	$48.84\pm 1.39\%$
		FF			
water	all	$86.89\pm 0.28\%$	$86.72\pm 0.07\%$	$83.62\pm 0.36\%$	$83.52\pm 0.30\%$
	gluteus	$87.79\pm 0.49\%$	$84.86\pm 2.31\%$	$78.17\pm 1.46\%$	$77.19\pm 3.77\%$
	psoas	$82.90\pm 0.29\%$	$81.01\pm 0.35\%$	$73.36\pm 0.64\%$	$72.85\pm 0.58\%$
	extensors	$98.64\pm 0.19\%$	$98.25\pm 0.16\%$	$97.64\pm 0.19\%$	$97.32\pm 0.11\%$
	adductors	$92.89\pm 0.47\%$	$90.92\pm 0.26\%$	$86.48\pm 0.28\%$	$85.60\pm 0.63\%$
fat	all	$88.21\pm 0.15\%$	$85.55\pm 0.25\%$	$84.28\pm 0.53\%$	$82.04\pm 0.22\%$
	gluteus	$94.03\pm 0.28\%$	$89.76\pm 0.25\%$	$84.10\pm 0.52\%$	$81.86\pm 0.61\%$
	psoas	$88.67\pm 0.15\%$	$87.76\pm 0.23\%$	$80.37\pm 0.60\%$	$80.99\pm 0.33\%$
	extensors	$98.78\pm 0.10\%$	$98.41\pm 0.12\%$	$98.03\pm 0.17\%$	$97.71\pm 0.09\%$
	adductors	$92.62\pm 0.38\%$	$90.25\pm 0.03\%$	$86.10\pm 0.61\%$	$84.26\pm 0.81\%$

In all cases a moderate performance decrease can be observed between the performance of the ROC curves and AUC scores on training data and on the unseen validation data. Despite varying amounts and selections of features across different folds, only minor variations in the prediction performance were seen between them. The performance, was similar for features based on the water and fat contrast alike. Thus, similar true positive rates are achieved for respective false positive rates on both contrasts. In addition, the ranking scheme further reduces the amount of selected features with only a minor performance drop in AUC scores. In most cases the derived mean AUC values were slightly higher when relying on fat texture features than water texture features. For the auxiliary targets, age was most difficult to predict, seen by more flat curves and lower AUC values, with FF being the easiest to estimate. The ROC curves also varied the most for FF between different muscles. When using all muscles, the training performance in AUC was lower compared to forests using features specifically selected for individual muscle groups. Nonetheless, the performance decrease on the validation sets were less drastic. For the mean multi-class accuracy performance, similar trends to the ROC curves and AUC scores are observed. We see that a large performance difference is present between estimating FF, BMI and age subgroups, with the latter being significantly more difficult given solely the selected features. As expected, predicting the right subgroup on the validation subset again shows a lower performance compared to the training set. In general, differences, e.g. between the selection of features based on all muscles and a specific muscle as can be seen for age and BMI, are more pronounced, compared to the respective AUC scores. In all cases the accuracy lies well above the accuracy of classifying the subgroup of the respective muscle by chance (of $16.67\%$ for six and $14.29\%$ for seven subgroups).

## Discussion

Radiomics are an important means to analyzing large cohort data quantitatively. Recently, radiomics have been applied to extract and leverage quantitative CT [17, 43], or MRI [44] texture features to analyze imaging data at large scales. It has also been shown to be extensible to multi-parametric studies [45] or be applicable for the classification of dental artifacts [46]. Motivated by these achievements, we proposed a feature extraction and selection pipeline from raw data to radiomics features of muscle tissue depicted in a two-point Dixon MRI sequence. Despite focusing on predominantly healthy tissue instead of tumors as in most aforementioned studies, we showed that selected features of the GNC cohort data [33] adhere to important conditions while retaining significant differences between subgroups of auxiliary information (targets). The workflow provides a distinctive ranking of texture features based on multiple or individual targets. We investigated differences of the selected features with respect to available auxiliary targets, including age, BMI and a muscle dependent FF. We illustrated and reported statistical differences between binned subgroups as well as differences between texture feature values of male and female subjects. Future investigations will be focused on applying the pipeline to MRI cohorts where patients with varying degree of sarcopenia have been quantified and labeled (categorized) to study differences in selected radiomic features and their value as biomarkers. For example, features that enabled a strong predictive quality for the FF (or BMI) may be of aid in reflecting changes in the fat infiltration in muscles and thereby indicate early onsets of sarcopenia.

Sarcopenia and the associated muscle weakness is very often responsible for the need for assistance of the affected patients. It affects a large proportion of the elderly population. It is known that sarcopenia occurs much more frequently and in earlier stages of life in the presence of internal diseases such as rheumatoid arthritis, metabolic disorders such as diabetes and malignant diseases. Imaging may provide new impulses for the differentiation of various forms of sarcopenia and for the monitoring of treatment measures.

The processing pipeline itself relies on several generation and selection steps, each of which could be replaced by alternative algorithms or heuristics. At this point, the focus of this work lies on the establishment of one variant of such a pipeline for large cohort data. We did not investigate e.g. alternative selection and prediction models that have been established for radiomics features [29], or other machine learning models such as a support vector machine or extreme gradient boosting [11] for CT images. The pipeline could also be extended to incorporate multi-scale information [45] to incorporate tissue characteristics at different scales. While performance differences can occur due to the alterations of methods in the pipeline, we focused on the ability to select features based on the given auxiliary targets as a whole and showed that the remaining features adhere to our defined conditions and retained distinctive information.

In our experiments, the diverse selection of the lowest ranked features, which includes features from different established feature subgroups (first order, glcm, and more) as well as different variants (original, LoG, wavelet), indicates that there are multiple promising feature candidates available for potential downstream tasks like further analysis of muscle tissues. The selection is also sensitive to the imaging contrast used for feature extraction. The selection is sensitive to variations in the folds as well as the auxiliary target and the included muscle groups. This can cause different amounts of selected features. Nonetheless, the range of amounts after all selection steps were close to their mean amounts. In addition, the selection sets share a large amount of common features as can be seen by the amounts of unique features across all folds in close relation to the lowest amount in most cases. Notably, the gluteal muscles seemed to incorporate the most complex texture patterns and variations as can be seen by the higher mean amount of selected features relative to other muscles for age and BMI.

Further, we identified features that score a low rank consistently with regards to multiple auxiliary targets. Based on the cross-validation procedure a repeatable and robust selection was further promoted. This provides us with a limited set of candidates for further investigations that can be tested for a regression or classification performance on non-imaging variables. For our overall post-selection radiomics feature ranking, we saw that the water contrast provided a more orderly ranking with close mean and median values and narrow boxplots. This indicates that the selected features provide the ability to be more distinguishable with respect to all auxiliary targets and for all muscle groups (on average) compared to features of the fat contrast. The fat texture features still provide a strong ranking with prominent differences, but the ranking order may vary more greatly between selected muscles and selected auxiliary targets. We also saw, that the ranking produced robust results for three of the four muscle groups with a clear order with respect to the performance of the features averaged across three auxiliary targets. A robust ranking for all subjects of all folds could also be established for the age, BMI and FF. Thus, depending on the muscle group, or the auxiliary target or an overall objective, the ranking allows to identify texture features that depict the included characteristic best.

The subgroup analysis showed that boxplots of the top performing features contain statistical significant differences. Despite large overlaps between neighboring subgroups, which is expected for the pre-dominantly healthy muscle tissue, clear trends were visible in the mean and median values. This was also the case if texture features were selected for individual muscles and their respective targets. Due to the selection process these trends were visible for male and female subgroups alike, making the features applicable independent from the sex of the subject. The vast majority of the lowest ranked features showed statistically significant differences between their subgroups for the joint processing of male and female subjects. Differences between corresponding male and female subjects were also present. However, not all differences were statistically significant in this case, which was more prevalent for features of the fat contrast. This variety allows for a further task dependent selection of features that are more or less sensitive to the respective gender differences. In addition, it was shown that a classification random forest predicting the respective subgroup bins was able to achieve strong AUC scores and moderate to high accuracy values on validation data based on selected features. This was the case for both contrasts as well as all three auxiliary targets.

We acknowledge several limitations in this study. The texture feature extraction and selection as well as subsequent analyses in this work focused on averaged voxel-wise features across each individual muscle volume. The investigations could be extended to more granular aggregation schemes with a focus on group-specific sub-regions. Additionally, the texture feature extraction parameters could be varied, to further analyze its impact on the repeatability and robustness of the result feature ranking. Investigations could also be performed with respect to the origin of the variability present in the ranking results for some features for certain muscles and auxiliary targets. Furthermore, analysis with respect to suitable tasks or conditions, such as sarcopenia, are planned to evaluate the robustness and variations of identified features with respect to selected cases and their externally measured non-imaging-based targets.

## Conclusion

In this preliminary study we showed that a pipeline for the extraction, selection and ranking of robust and distinctive radiomics features of muscle tissue from Dixon-weighted whole-body MR imaging raw data can be established for the analysis in large cohort studies. Future work remains to investigate the applicability of the identified features for the analysis of intricate degenerative characteristics in different muscle tissues.

## Data Availability

The data that support the findings of this study are available from the German National Cohort (GNC) (https://www.nako.de) but restrictions apply to the availability of these data. Data access applications can be submitted via NAKO TransferHub (transfer.nako.de/transfer/index). All codes of this study are made publicly available: https://github.com/lab-midas/muscle_texture.
